# Optimization of SABRE for polarization of the tuberculosis drugs pyrazinamide and isoniazid

**DOI:** 10.1016/j.jmr.2013.09.012

**Published:** 2013-12

**Authors:** Haifeng Zeng, Jiadi Xu, Joseph Gillen, Michael T. McMahon, Dmitri Artemov, Jean-Max Tyburn, Joost A.B. Lohman, Ryan E. Mewis, Kevin D. Atkinson, Gary G.R. Green, Simon B. Duckett, Peter C.M. van Zijl

**Affiliations:** a Russell H. Morgan Department of Radiology and Radiological Science, The Johns Hopkins University School of Medicine, Baltimore, MD, USA; b F.M. Kirby Research Center for Functional Brain Imaging, Kennedy Krieger Institute, Baltimore, MD, USA; c Bruker BioSpin GmbH, Silberstreifen, Rheinstetten, Germany; d Bruker UK Limited, Banner Lane, Coventry, UK; e Centre for Hyperpolarisation in Magnetic Resonance, University of York, Heslington, York, UK

**Keywords:** SABRE, Parahydrogen, Hyperpolarization, Pyrazinamide, Isoniazid, Mycobacterium Tuberculosis

## Abstract

Hyperpolarization produces nuclear spin polarization that is several orders of magnitude larger than that achieved at thermal equilibrium thus providing extraordinary contrast and sensitivity. As a parahydrogen induced polarization (PHIP) technique that does not require chemical modification of the substrate to polarize, Signal Amplification by Reversible Exchange (SABRE) has attracted a lot of attention. Using a prototype parahydrogen polarizer, we polarize two drugs used in the treatment of tuberculosis, namely pyrazinamide and isoniazid. We examine this approach in four solvents, methanol-d_4_, methanol, ethanol and DMSO and optimize the polarization transfer magnetic field strength, the temperature as well as intensity and duration of hydrogen bubbling to achieve the best overall signal enhancement and hence hyperpolarization level.

## Highlights

• SABRE polarization of two drugs.
• Up to 8% polarization of proton is achieved in less than 1 min.
• SABRE polarization dependence on polarization field, temperature and solvent is determined.

## Graphical abstract

**Figure:**
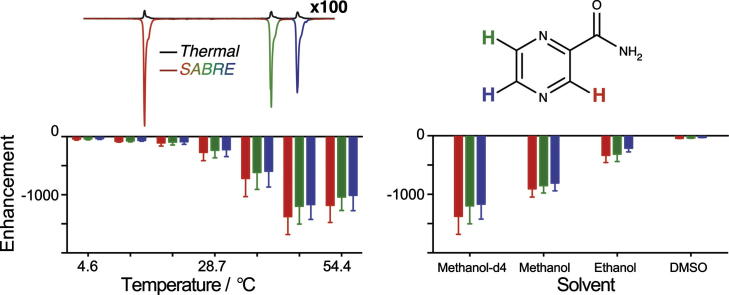

## 1 Introduction

By producing nuclear spin polarization far beyond that available at thermal equilibrium, hyperpolarization can provide improved sensitivity for NMR, enabling the detection of less concentrated molecules. In the area of molecular imaging, MRI has recently been used to study the distribution [1] and metabolism [2–4] of hyperpolarized substrates. For instance, multiple studies have reported on the conversion of hyperpolarized ^13^C-labeled pyruvate to its metabolic products, alanine, lactate and carbonate *in vivo* [2–6], in which higher lactate production is an important indicator of cancer. This technique is already being translated to the clinic and a first trial is ongoing [7].

Major hyperpolarization techniques include dynamic nuclear polarization (DNP) [8,9], spin exchange optical pumping polarization of noble gases [10] and parahydrogen induced polarization (PHIP) [11–16]. Parahydrogen is a spin isomer of hydrogen with an antisymmetric singlet spin state. By incorporating this pure spin state into a molecule through a hydrogenation reaction, large signal enhancements have been observed in a variety of situations as first conceived by Bowers and Weitekamp [12] and Pravica and Weitekamp [14]. In 2009, Duckett’s group developed a parahydrogen polarization technique that works without the need for the chemical modification of the substrate [17]. In this approach, the substrate and the parahydrogen bind to a catalyzing metal complex simultaneously, thus enabling polarization to be transferred to the substrate through the scalar coupling network. The polarized substrate is subsequently released, and replaced by new substrate which is polarized in turn. Such Signal Amplification By Reversible Exchange (SABRE) has already been applied to detect trace amounts of chemicals [18–20] and used in conjunction with zero-field NMR spectroscopy [21].

According to a theoretical description of SABRE, the signal enhancement level depends on the binding kinetics and the magnetic field in which polarization transfer occurs [22]. In order to achieve better enhancement, new catalyst precursors have been developed to tune the binding kinetics. Enhancements can be boosted by using the bulky electron-donating phosphines of the Crabtree catalyst [23]. Currently, an iridium N-heterocyclic carbene complex [24] shows the highest polarization transfer efficiency.

**Fig. 1:**
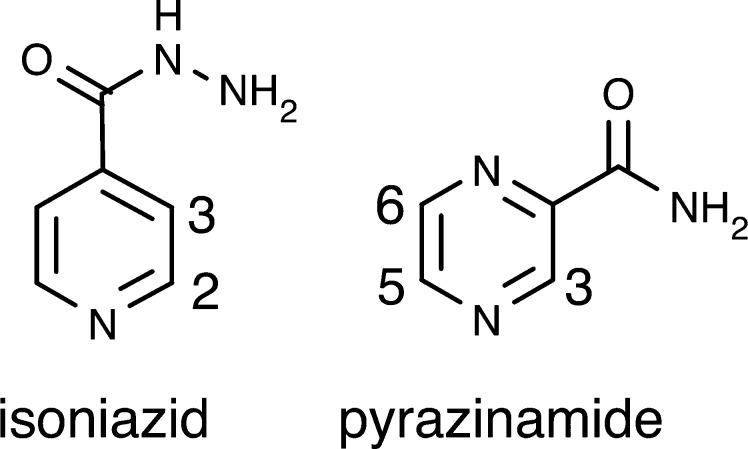
Chemical structure of isoniazid and pyrazinamide.

In this paper, we investigated the SABRE polarization of two drugs that are used clinically, isoniazid and pyrazinamide [25]. Isoniazid treats tuberculosis meningitis, and pyrazinamide is used in combination with other drugs in the treatment of Mycobacterium tuberculosis. Isoniazid is a pyridine derivative, and pyrazinamide is a pyrazine derivative. They are nitrogen containing heterocyclic aromatic organic compounds (Fig. 1) and are thus able to bind to the iridium atom of the catalyst precursor. Therefore, they are suitable for SABRE polarization.

In previous work, methanol-d_4_ was used as a solvent for SABRE polarization, which is not suitable for injection into small animals. In this paper, we therefore also investigated the possibility of SABRE polarization in solvents more suitable for *in vivo* applications, namely DMSO and ethanol.

The enhancement efficiency depends on the polarizing magnetic field and temperature as well as on the hydrogen bubbling intensity and time. These conditions were optimized for each solvent.

## 2 Experimental section

### 2.1 Sample preparation

The samples used for the SABRE experiments contained 0.40 mM of the catalyst precursor [Ir(COD)(IMes)Cl] [COD = cyclooctadiene, IMes = 1,3-bis(2,4,6-trimethylphenyl)imidazole-2-ylidene] and 4.0 mM of the selected substrate, either isoniazid or pyrazinamide (Sigma–Aldrich, St. Louis, MO). This catalyst to substrate ratio of 1:10 was chosen following Ref. [26]. The solvents were methanol-d_4_ (Cambridge Isotope Laboratories, Andover, MA), methanol, ethanol and dimethyl sulfoxide (DMSO) (Sigma–Aldrich, St. Louis, MO). The total sample volume was 3.5 mL.

### 2.2 SABRE polarization

Parahydrogen was prepared using a parahydrogen generator that cools the hydrogen gas to 36 K in the presence of a metal catalyst, after which the fraction of parahydrogen becomes 92.5%. Subsequently, the sample containing the substrate and the catalyst precursor was loaded into a mixing chamber positioned underneath the magnet of a Bruker 700 MHz spectrometer. The temperature of the sample was controlled by a home-built water bath system. Polarization was achieved by bubbling parahydrogen through the sample. The sample was then pneumatically transferred to the flow cell in the spectrometer. This process took about 2 s. Once the sample was in the NMR probe, spectra were acquired immediately. After data acquisition, the sample was returned to the mixing chamber for repolarization.

### 2.3 NMR measurement

In experiments using methanol-d_4_ as a solvent, NMR spectra were acquired after a *π*/2 hard pulse. When non-deuterated solvents were used, solvent suppression was achieved using excitation sculpting pulse sequences [27]. The shaped pulses were 20 ms Gaussian pulses that excite all of the solvent peaks.

## 3 Results

### 3.1 Pyrazinamide

**Fig. 2:**
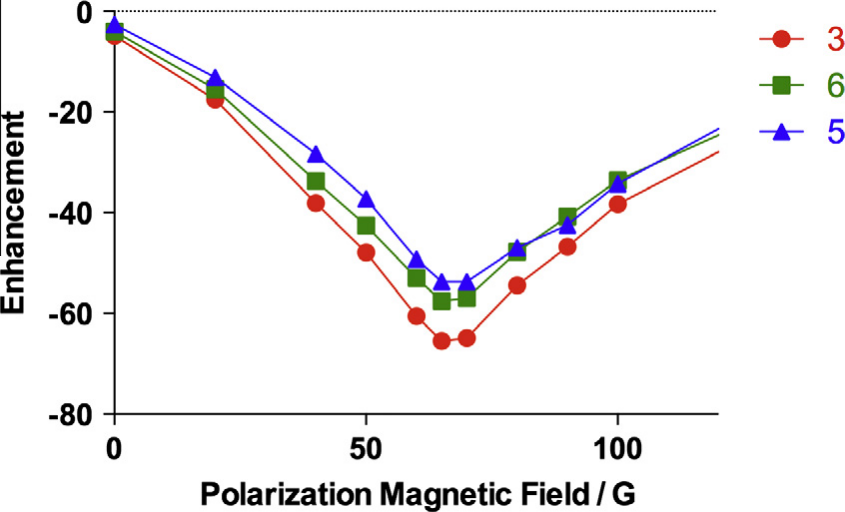
Enhancement of pyrazinamide protons as a function of polarization magnetic field strength in methanol-d_4_ at room temperature.

The total magnetic field of the sample in the preparation chamber is the vector summation of the stray field of the scanner magnet and the magnetic field generated by a small electromagnetic coil surrounding the sample, which is tunable up to ±145 G. The mixing chamber is placed directly underneath the NMR magnet in a region where the stray field is vertical so that it can be completely compensated to achieve a zero overall magnetic field in the sample. According to the SABRE mechanism, optimum enhancement occurs when the differences in resonance frequency of the protons are of the same order as the scalar couplings [22]. While the optimal polarization field cannot be predicted straightforwardly, it can be easily determined experimentally by varying the magnetic field with the small coil around the sample. Fig. 2 shows the dependence of the enhancement of pyrazinamide on local magnetic field strength in methanol-d_4_ at room temperature.

In the range of 0–120 G, the signal enhancement for all the three aromatic protons of pyrazinamide was always of the same order of magnitude and negative. The shape of the dependency of the enhancement was a “V” curve with a maximum absolute enhancement at 65 G, which is very close to the value 70 G reported by Cowley et al. for pyridine [24].

Subsequently, the parameters for the hydrogen bubbling were tested at the optimal magnetic field of 65 G. The mixing of hydrogen gas with the catalyst precursor and substrate in liquid phase, which is required by the SABRE mechanism, was achieved by bubbling the hydrogen gas through a porous ceramic rod. This bubbling was controlled by the input and output pressure of parahydrogen in the mixing chamber. Usually, a larger pressure difference meant more intense bubbling. However, a very large bubble size produced by a pressure difference that is too large should be avoided. The hydrogen bubbling time should be long enough to ensure complete reaction of hydrogen, substrates and the catalyst. In our case, we increased the hydrogen bubbling time until the polarization stopped increasing. These timing and pressure parameters were solvent dependent (Table 1).

**Fig. 3:**
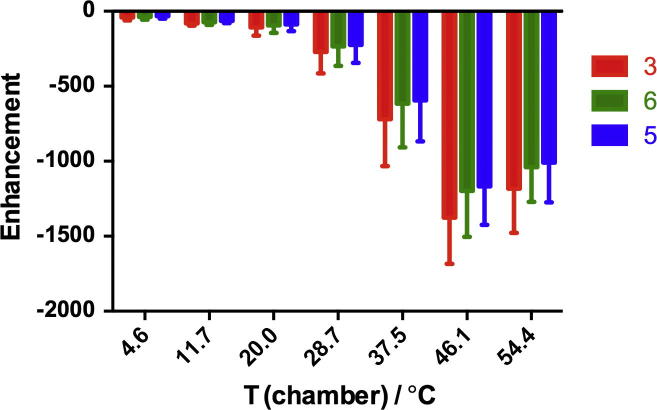
Enhancement levels for pyrazinamide ^1^H NMR signals in methanol-d_4_ revealing a dependence on polarization temperature for transfer in a magnetic field of 65 G.

The temperature dependency was also investigated. For the polarization of pyrazinamide in methanol-d_4_ in a magnetic field of 65 G, the enhancements (Fig. 3) of all three protons were relatively low for temperatures below 20.0 °C. From 20.0 to 46.1 °C, the enhancements of all three protons increased dramatically, before leveling off.

**Fig. 4:**
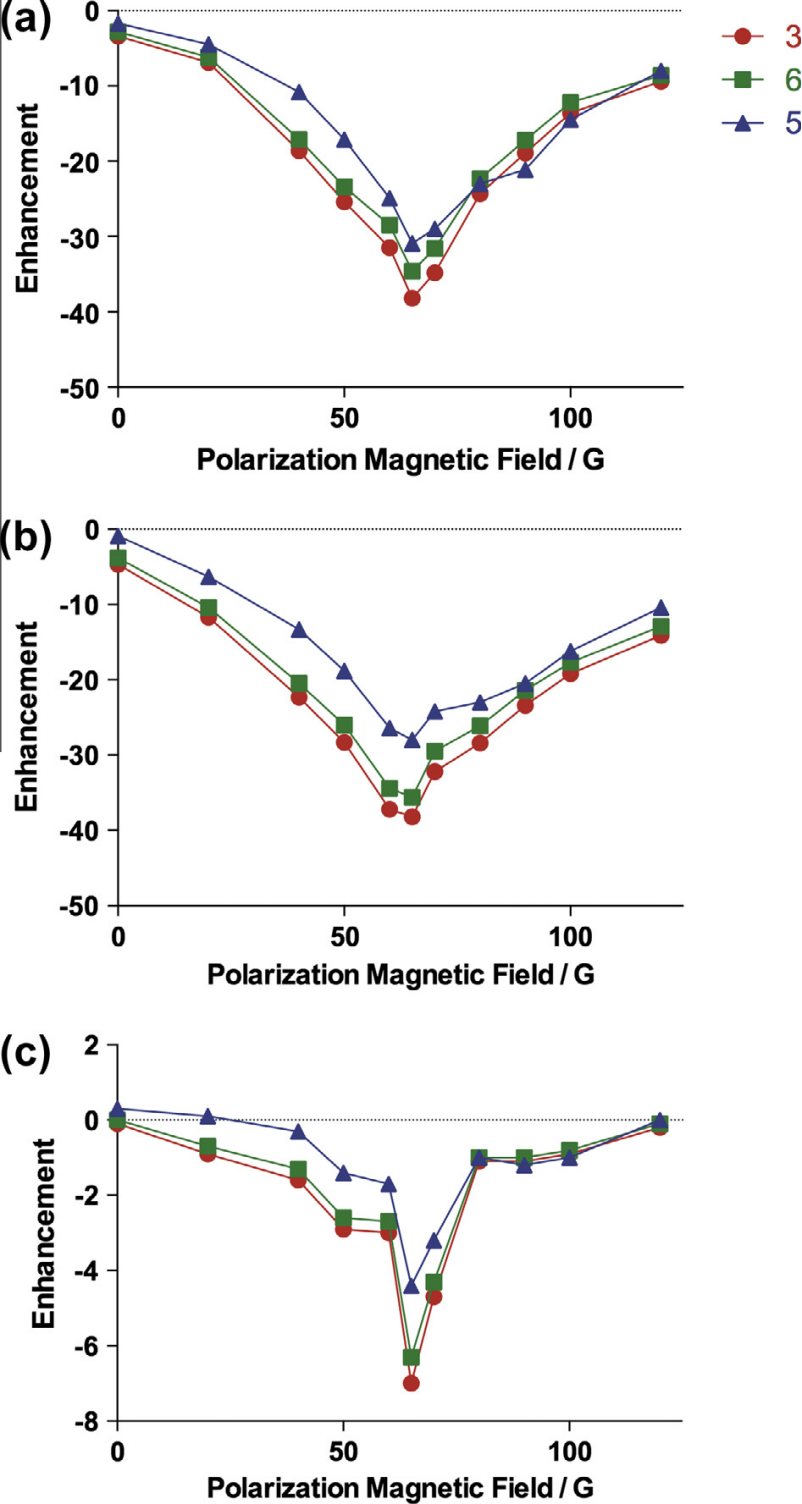
Polarization transfer field dependence of the observed ^1^H signal enhancement of pyrazinamide in (a) methanol, (b) ethanol and (c) DMSO at room temperature.

Methanol-d_4_ was chosen as the first test solvent based on the literature [17,20,23,24]. Methanol was also investigated and found to give enhancements only slightly lower than its deuterated analog (Fig. 4). Two other solvents, ethanol and DMSO, were chosen because of their lower toxicity and suitability for intravenous injection for study *in vivo*. DMSO is often used as a drug vehicle in medical research. Water was not considered as a solvent due to the catalyst precursor being insoluble. The polarization field dependencies for pyrazinamide in these other solvents showed patterns similar to methanol-d_4_, with optimal enhancement at 65 G. While the enhancement in ethanol resembled that in methanol, it was about an order of magnitude smaller in DMSO (Fig. 4).

**Fig. 5:**
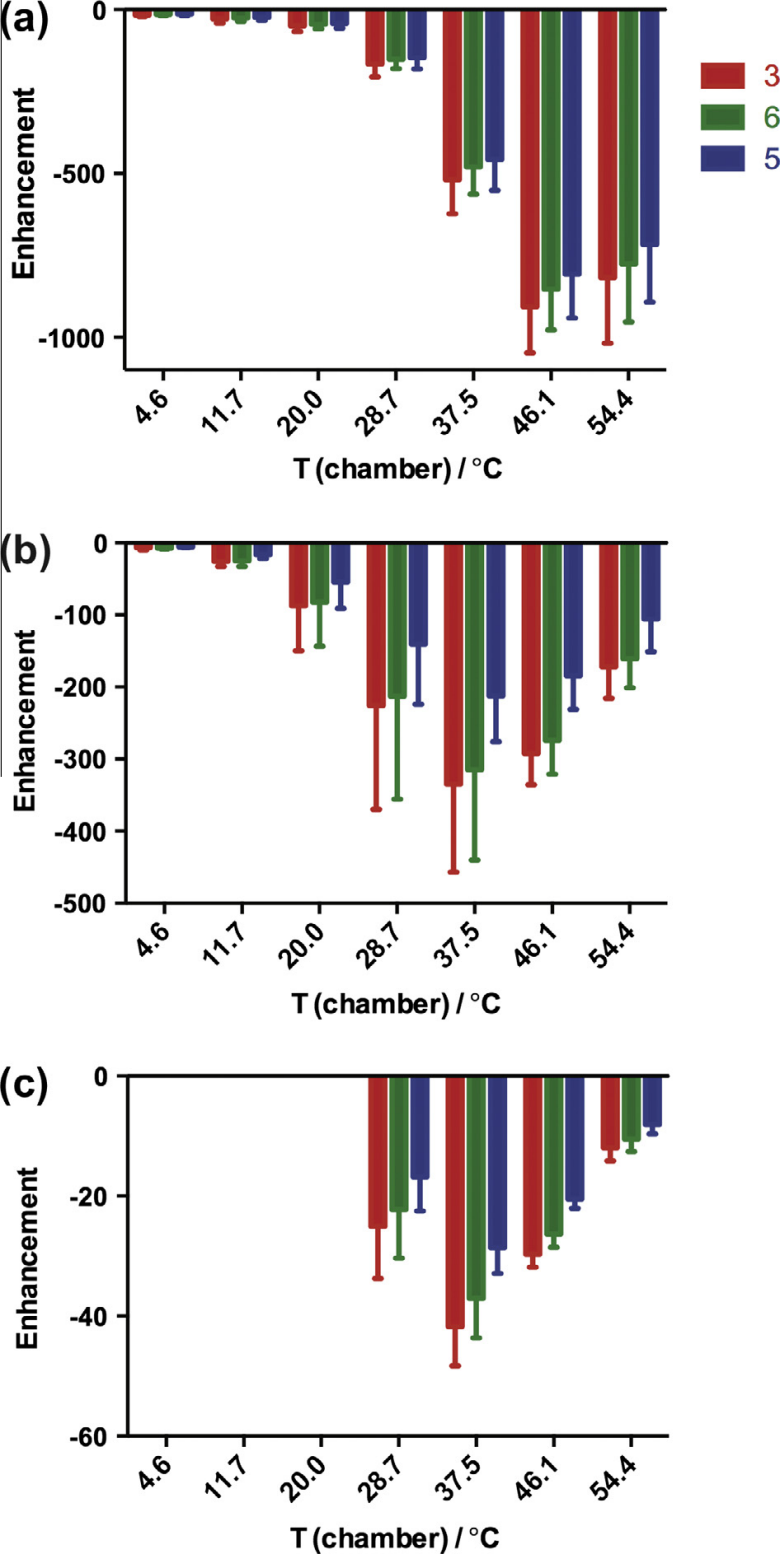
Dependence of the signal enhancement of pyrazinamide on polarization temperature in methanol (a), ethanol (b) and DMSO (c) in a magnetic field of 65 G. Notice the different vertical scales.

The enhancement dependencies on temperature in these three solvents at 65 G are plotted in Fig. 5. The polarization studies in DMSO were only carried out at higher temperatures because it was difficult to transfer the sample when it is too viscous, which occurs at a temperature close to the freezing point of the solvent (DMSO, 19 °C).

Compared to methanol-d_4_, the enhancements in methanol were reduced to a half and in ethanol to a quarter, while those in DMSO were an order of magnitude smaller and thus less suitable to polarize pyrazinamide.

### 3.2 Isoniazid

**Fig. 6:**
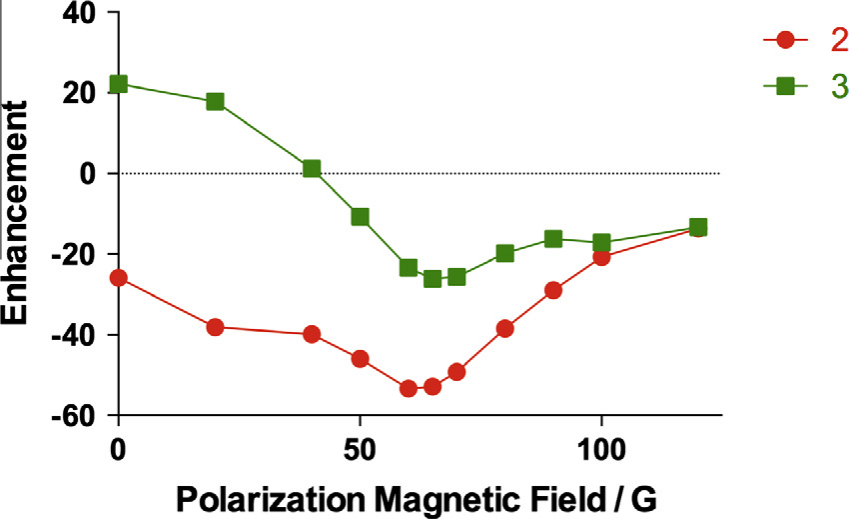
Dependence of enhancement of isoniazid in methanol-d_4_ on polarization magnetic field strength at room temperature.

In the case of isoniazid, the enhancements of the two protons again showed a “V-curve” dependency on polarization magnetic field (Fig. 6). Interestingly, at 0 G, the polarization of proton 2 was negative while that of proton 3 was positive. The optimal magnetic field for both protons was again very similar, namely around 60–65 G. A magnetic field of 65 G was therefore again chosen to study the temperature dependence. At this field strength, the polarization of protons was almost twice of that of proton 3, probably due to proton 2 being closer to the nitrogen atom, which directly bonds to iridium upon ligation.

**Fig. 7:**
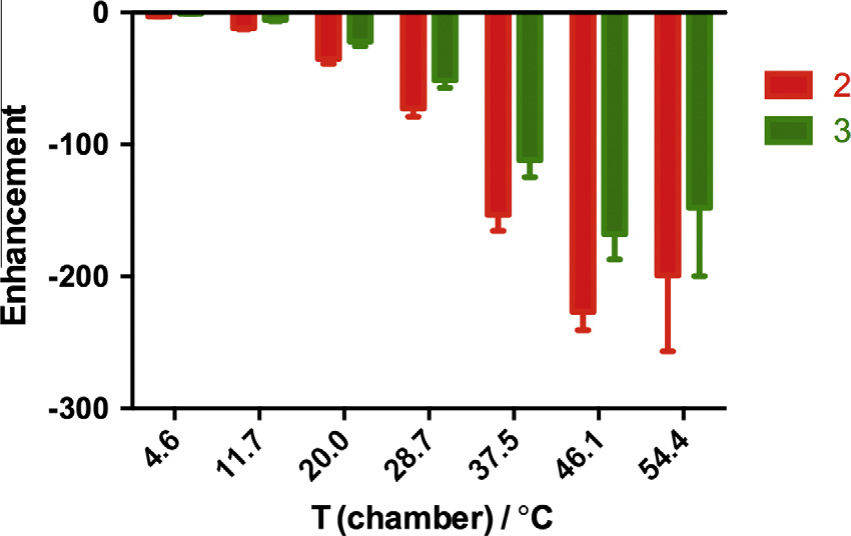
Dependence of the signal enhancement observed for isoniazid in methanol-d_4_ on polarization temperature in a magnetic field of 65 G.

The polarization of isoniazid in methanol-d_4_ at a magnetic field of 65 G was measured over the temperature range 4.7–54.4 °C (Fig. 7). The signal enhancements observed for both protons increased with temperature until reaching a maximum enhancements of −220 and −150 fold at 46.1 °C. At higher temperature (54.4 °C), the enhancements were slightly decreased.

**Fig. 8:**
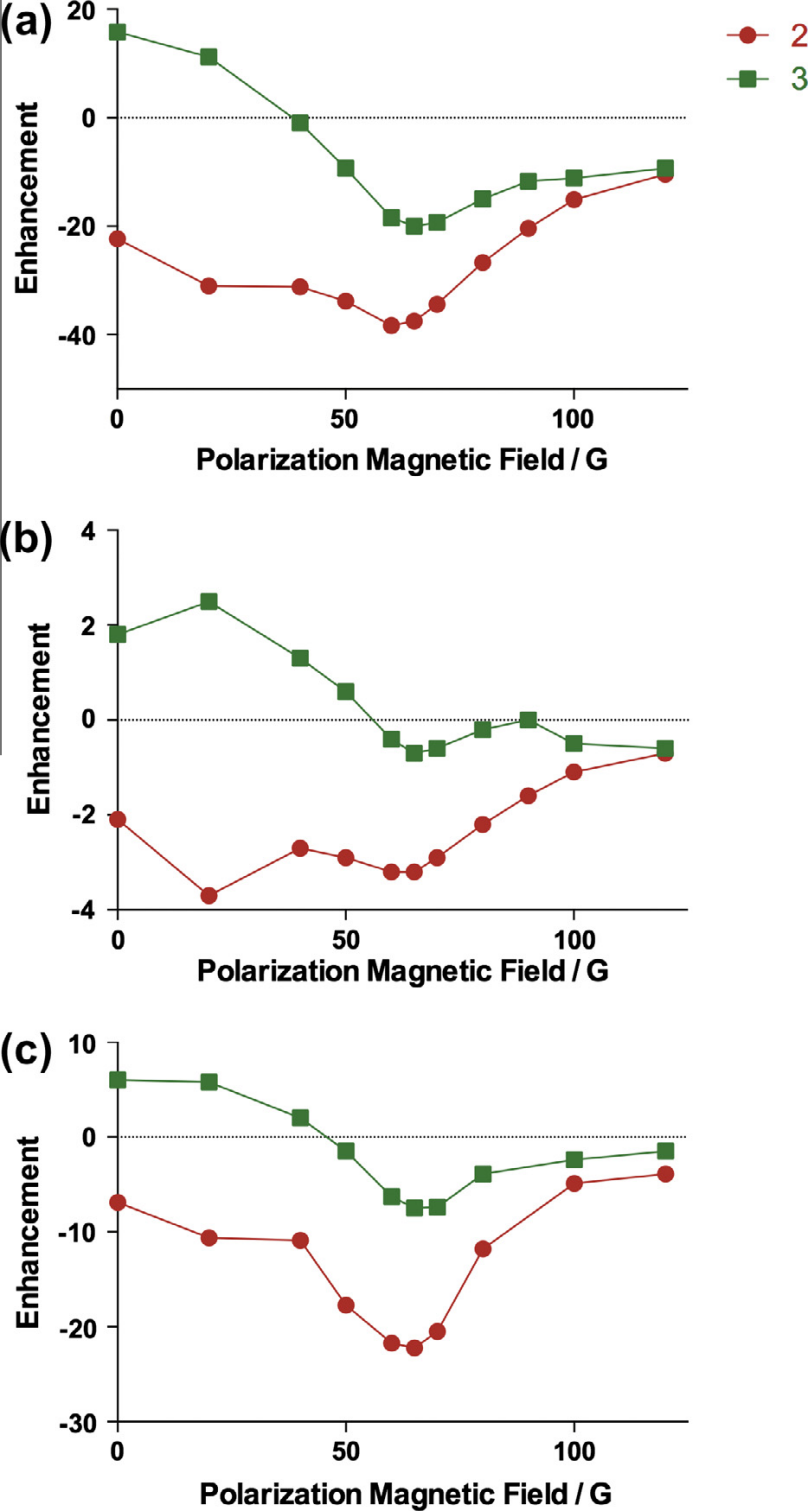
Polarization transfer field dependence for the ^1^H signal enhancement of isoniazid in (a) methanol, (b) ethanol at room temperature, and (c) DMSO at 37.5 °C.

**Fig. 9:**
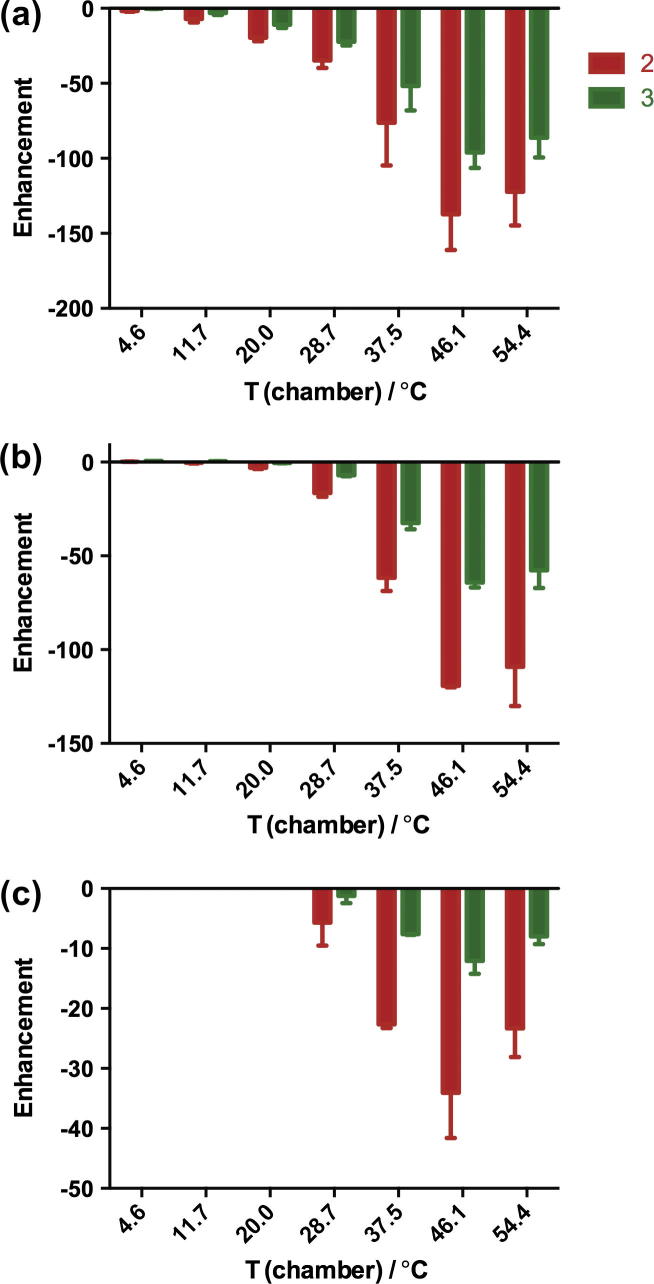
^1^H NMR signal enhancement of pyrazinamide in methanol (a), ethanol (b) and DMSO (c) dependence on polarization temperature in a magnetic field of 65 G. Notice the different vertical scales.

The polarization of isoniazid in the other three solvents was also investigated for a polarization transfer magnetic field of 65 G (Fig. 9), even though this magnetic field was not optimal for the polarization in ethanol at room temperature (Fig. 8). The best enhancements were always at 46.1 °C. The SABRE enhancement of isoniazid shows similar solvents dependence as that of pyrazinamide. Compared to methanol-d_4_, the enhancements in methanol were slightly lower, in ethanol about a half, and in DMSO about one fifth, making it a less suitable solvent in which to polarize isoniazid via SABRE.

## 4 Discussion

According to SABRE theory [22], polarization transfer, binding kinetics and spin relaxation determine the size of the enhancement. The polarization of parahydrogen is transferred to the substrate through *J* coupling networks, the strength of which is determined by the chemical structure and bonding strength of the substrate-metal complex. Since the multi-bond *J* couplings between the parahydrogen and the substrate are small, a relative long residence time on the metal (in the order of 100 ms to s) is required for effective transfer. Thus, in the case of fast binding kinetics, the short lifetime of the substrate-metal complex will decrease SABRE enhancements. On the other hand, since the concentration of the substrate is much larger than that of the catalyst precursor, polarization of all of the substrate molecules requires relative fast exchange between the substrate in free form and metal bound form. During the exchange, the spin relaxation decreases the polarization. Therefore, binding kinetics that is too slow does not lead to significant SABRE enhancements either.

These data show a general trend that the enhancements are better at higher temperature than at room temperature. Generally, the binding kinetics and molecular tumbling are faster at higher temperature. Also, the spin relaxation rates are smaller at higher temperature for these small molecules in the extreme narrowing limit. Faster binding kinetics and slower relaxation lead to higher enhancements, with the best enhancement in most cases occurring at 37.5–46.1 °C.

The enhancements are negatively correlated with the viscosity of the solvents (methanol < ethanol < DMSO). In the extreme narrowing limit, proton spin relaxation rates are faster for the substrate-metal complex in more viscous solvents, causing polarization loss and a concomitant lower SABRE enhancement. By replacing the protons with deuterons, the spin relaxation reduces and methanol-d_4_ showed the best enhancement.

In pyrazinamide the parahydrogen spin order is shared with three protons, while it is shared with four in isoniazid. One might therefore expect that for equivalent transfer efficiency the levels of signal enhancement would be 4:3. Given the 1400:230 ratio, we conclude that pyrazinamide reflects a better spin system. SABRE enhancement requires the complexation of the substrate of the catalyst precursor, which is through the formation of a chemical bond between the iridium and the nitrogen in the aromatic ring. Effective polarization transfer requires strong *J* coupling. In the substrate metal complex, the polarization can be transferred to proton 2 in isoniazid and all three aromatic proton in pyrazinamide through a 4-bond *J* coupling. However, the transfer to proton 3 in isoniazid is through a much smaller 5-bond *J* coupling. This is the probable cause of the much smaller enhancement of proton 3 compared to that of proton 2 in isoniazid. In addition, pyrazinamide has two nitrogen atoms in the aromatic ring, both of which are able to bind to iridium. This is one possible reason that the enhancement for pyrazinamide is much higher than that of isoniazid.

## 5 Conclusion

We report the polarization of two drugs via SABRE that are used clinically for treating tuberculosis, pyrazinamide and isoniazid [25]. To achieve the best enhancement level, the strength of the polarizing magnetic field and temperature were optimized together with the bubbling of parahydrogen. Using a fixed catalyst-to-substrate ratio of 1:10, the best enhancements for all three protons in pyrazinamide were obtained in a polarizing magnetic field of 65 G for all solvents. In all solvents, the enhancements at higher temperature were better than that at room temperature. In methanol-d_4_, up to −1400 times enhancement was obtained, corresponding to 8% polarization, which is comparable to that of DNP [28–30]. In methanol, ethanol and DMSO, the best enhancements were −960, −320 and −40 respectively. For isoniazid, the best enhancements in methanol-d_4_, methanol, ethanol and DMSO were −230, −140, −120 and −34 respectively in a magnetic field of 65 G. The enhancement of proton 3 was only 50–70% of that of proton 2.

Both systems show a similar temperature profile where 37.5–46.1 °C seems to reflect the optimum temperature and hence lifetime of the polarization transfer catalyst. It would therefore appear that the *J*-coupling framework in the pyrazinamide system is more suited for optimal transfer. Considering the solvent effects, the SABRE enhancement can be increased by minimizing the spin relaxation of the substrate-metal complex, namely using less viscous solvent and deuterated solvent.

## Figures and Tables

**Table 1:** Parahydrogen bubbling parameters for the four solvents used in the paper.

Solvent	Forward pressure (bar)	Backward pressure (bar)	Bubbling time (s)
Methanol-d_4_	2.5	1.0	30
Methanol	2.5	1.0	10
Ethanol	2.0	0.0	10
DMSO	4.9	0.0	50
